# Evolution of Wenger's concept of community of practice

**DOI:** 10.1186/1748-5908-4-11

**Published:** 2009-03-01

**Authors:** Linda C Li, Jeremy M Grimshaw, Camilla Nielsen, Maria Judd, Peter C Coyte, Ian D Graham

**Affiliations:** 1 Department of Physical Therapy, University of British Columbia, Arthritis Research Centre of Canada, Vancouver, Canada; 2 Ottawa Health Research Institute, Clinical Epidemiology Program, Centre for Best Practice, Institute of Population Health, University of Ottawa, Ottawa, Canada; 3 Centre for Health Technology Assessment, National Board of Health, Copenhagen, Denmark; 4 Canadian Health Services Research Foundation, Ottawa, Canada; 5 Department of Health Policy, Management and Evaluation Faculty of Medicine, University of Toronto, Toronto, Canada; 6 Canadian Institutes of Health Research; School of Nursing, University of Ottawa, Ottawa, Canada

## Abstract

**Background:**

In the experience of health professionals, it appears that interacting with peers in the workplace fosters learning and information sharing. Informal groups and networks present good opportunities for information exchange. Communities of practice (CoPs), which have been described by Wenger and others as a type of informal learning organization, have received increasing attention in the health care sector; however, the lack of uniform operating definitions of CoPs has resulted in considerable variation in the structure and function of these groups, making it difficult to evaluate their effectiveness.

**Objective:**

To critique the evolution of the CoP concept as based on the germinal work by Wenger and colleagues published between 1991 and 2002.

**Discussion:**

CoP was originally developed to provide a template for examining the learning that happens among practitioners in a social environment, but over the years there have been important divergences in the focus of the concept. Lave and Wenger's earliest publication (1991) centred on the interactions between novices and experts, and the process by which newcomers create a professional identity. In the 1998 book, the focus had shifted to personal growth and the trajectory of individuals' participation within a group (i.e., peripheral versus core participation). The focus then changed again in 2002 when CoP was applied as a managerial tool for improving an organization's competitiveness.

**Summary:**

The different interpretations of CoP make it challenging to apply the concept or to take full advantage of the benefits that CoP groups may offer. The tension between satisfying individuals' needs for personal growth and empowerment versus an organization's bottom line is perhaps the most contentious of the issues that make CoPs difficult to cultivate. Since CoP is still an evolving concept, we recommend focusing on optimizing specific characteristics of the concept, such as support for members interacting with each other, sharing knowledge, and building a sense of belonging within networks/teams/groups. Interventions that facilitate relationship building among members and that promote knowledge exchange may be useful for optimizing the function of these groups.

## Introduction

A major challenge to integrating evidence into practice is that it involves a complex process of acquiring and converting both explicit and tacit knowledge into clinical activities. Explicit knowledge is codified information such as peer-reviewed articles, rules, and guidelines, which can be readily shared among people. However, to apply this knowledge in practice, practitioners must make sense of the concrete information in the context in which it is used. This process of establishing meaning can be facilitated by discussions with colleagues and mentors or by observing how others apply the knowledge and then try it themselves [1-4]. As a result, we see a growing number of informal groups and networks that create opportunities for knowledge exchange. Communities of practice[5,6] (CoPs) (the concept itself is referred to as 'community of practice'), which have been described as a type of informal learning organization, are gaining popularity in the health sector [7-10]. A recent (October 2008) Google search on the exact phrase *Health *'*Communities of Practice*' yielded over 213,000 hits.

CoPs have been used in the education and business sectors for over 20 years[5], but their use in the health care field has been limited and their structures are generally inconsistent. Some of these groups resemble informal networks, where the goal and structure of the group tend to be loosely defined[5], and others are similar to support groups, where the main goal is to enhance self-efficacy[11]. Some researchers even argue that a CoP is analogous to a well-run network[12] or a multidisciplinary team[13]. The lack of consistency in the interpretation of the CoP concept makes it difficult to describe, develop, and measure the effectiveness of a CoP. In this paper, we discuss CoPs in the context of learning communities. We trace and explain the evolution of Wenger's CoP concept and illustrate the challenges of applying the concept given the divergences of its central focus. Our goal is to indentify promising directions to advance the use of the CoP concept in the health care setting.

## Methods

This work was conducted within a large research synthesis project that aimed to examine how CoPs were defined and used in the business and health sectors, and to evaluate evidence for the effectiveness of CoPs in the health sector in improving the uptake of best practices. The methodology and findings of the research synthesis are reported elsewhere[14] and are summarised in Table 1. The current paper focuses on the authors' interpretations of Wenger's germinal work and recommendations for future research to advance the understanding and use of the CoP concept.

**Table 1:** Description of communities of practice research synthesis project

Objectives:	• To examine how CoPs were defined and used in the business and health sectors.
	• To evaluate the evidence of CoPs in the health sector.
Search strategy:	• We searched the literature published between 1991 and 2005.
	• Database search: Medline, EMBASE, CINAHL, HealthSTAR, ERIC, ECONLIT, AMED, and ProQuest.
	• Hand-searched *Journal of Continuing Education in the Health Professions*, *Medical Education*, and *Harvard Business Review*.
Eligibility criteria	• Primary studies that involved groups, teams, or learning environments that were either labelled as CoPs or were developed using CoP and/or other related concepts (e.g., situated learning, legitimate peripheral learning) as the guiding framework.
Synthesis approach:	• Meta-narrative approach
	○ The research synthesis focused on:
	▪ The authors' interpretations of the CoP concept.
	▪ The key characteristics of CoP groups.
	▪ The common elements of CoP groups.
	• Meta-analysis to assess the effectiveness of CoPs in the health sector.
Search results:	• 1421 articles were obtained; of those, we found 13 primary studies from the health sector and 18 from the business sector.
Key findings:	• The structure of CoP groups varied greatly, ranging from voluntary informal networks to work-supported formal education sessions, and from apprentice training to multidisciplinary, multi-site project teams.
	• Four characteristics were identified from CoP groups:
	○ CoP members interact with each other in formal and informal settings.
	○ CoP members share knowledge with each other.
	○ CoP members collaborate with each other to create new knowledge.
	○ CoP groups foster the development of a shared-identity among members.
	• These characteristics, however, were not consistently present in all CoPs.
	• There was a lack of clarity in the responsibilities of CoP facilitators and how power dynamics should be handled within a CoP group.
	• We were unable to identify any studies that used experimental, quasi-experimental, or observational designs, and evaluated CoPs for improving health professional performance, health care organizational performance, professional mentoring, and patient outcome. Therefore, it was not possible to conduct a meta-analysis.

*CoPs = Communities of practice

We first came across Wenger's work when one of the authors (LL) searched the literature on knowledge translation and implementation and found an article in *Harvard Business Review *that described the use of CoP as a tool that could improve an organization's capacity to develop and share new knowledge[15]. Intrigued by the concept of CoP, we subsequently studied Wenger's major publications:

• Lave and Wenger (1991). *Situated Learning: Legitimate Peripheral Participation*[16].

• Wenger (1998). *Communities of Practice: Learning, Meaning and Identity*[17].

• Wenger, McDermott, and Snyder (2002). *Cultivating Communities of Practice*[5].

## Discussion

### Learning Communities and Communities of Practice

CoPs are considered to be a type of learning community[5,16,17]. In order to understand the CoP concept we must therefore first define 'community' and 'learning community.' 'Community' generally describes groups of people (e.g., a town, a school) connected by a common interest and who define their identities by the roles they play and the relationships they share in the group's activity[18]. A community can exist over time despite a change of participants. It develops its own culture and communication methods as it matures[18].

Social learning theorists suggest that communities provide a foundation for sharing knowledge. It is believed that individuals can learn by observing and modelling other people. Bandura[19] emphasizes that observing other people's behaviour allows for a safer and more efficient way of acquiring complex behaviours or skills than learning by trial and error. Social constructivists, such as Cobb and colleagues[20,21], understand learning as an individual's responsibility and the community is the means by which people learn. Communities provide a safe environment for individuals to engage in learning through observation and interaction with experts and through discussion with colleagues.

The term 'learning community' became popular among educators in the 1990s [22]. Graves emphasized the importance of social relationships between experts and learners, and the new roles assumed by all players[22]. For example, teachers were encouraged to step back from their usual role of expert, and to act instead as facilitators and co-participants who can display ignorance as well as knowledge. The equalization of roles between teachers and learners in a community often maximises the participation of everyone, but may also create a sense of discomfort and insecurity. Tension can arise among learners who are expected to work collaboratively, but are often evaluated individually, and thus competitively, on their performance and their ability to master the knowledge acquired. Some people may perceive these new roles as risky and uncomfortable, which may subsequently lead to less engagement. A learning community must therefore develop a high level of trust among participants in order to be functional[23].

Traditionally, members of a learning community reside in the same location[22]. However, as groups migrate and become less homogenous, configurations of 'group identity' based on geographic location become less appropriate. Nowadays communities are linked less by location and more by common interests and goals. Many new learning communities have developed as technology makes global communication increasingly easier and faster. E-mail discussion lists and online information management systems (e.g., the Blackboard [24]) have become popular communication tools for synchronized and asynchronized dialogues. Hence, virtual learning communities are more fluid than traditional communities[25].

Simply labelling a group of people as a learning community does not guarantee that it will function as one. A number of situations can hinder relationship building and the growth of communities. For example, tight bonds between members can become exclusive and thus present a major barrier to the integration of newcomers. Without proper monitoring, this closeness can hinder the acceptance of external input and the development of external collaborations[5]. A community can also become a clique when relationships among members are so strong that they overshadow all other concerns. There is also a risk of group-thinking, which can constrain individual growth and creativity if individual members are discouraged from standing out in a community. Furthermore, failure to accommodate change or variation can render a community dysfunctional; a community can become dormant if it fails to attract new members. All the above situations can hinder exchanges of information and the development of innovative ideas within the community. Finally, in the case of a virtual learning community, issues regarding privacy, user-friendliness of online technologies, and the ability to access a computer can become fatal barriers to an individual's ability to participate[25,26].

A strong learning community fosters interactions and relationships based on mutual respect and trust[6,15]. It creates a social structure for individuals to share ideas and artefacts (e.g., stories, documents, recordings) that support community activities and help individuals make sense of new knowledge. Newcomers in particular can benefit from having access to the archived material in addition to the experience of and mentoring from experts. These conditions provide a rich environment for individuals to share information and ways to apply new knowledge in practice.

### Evolution of the Germinal Work on Community of Practice

The elements of a learning community formed the basis for the development of the CoP concept in the early 1990s. Initially the concept aimed to provide a template for examining the learning that occurs among practitioners in a social environment[16], but the focus of the concept has diverged during subsequent years[27]. To understand CoP and to appreciate its various interpretations, one needs to revisit the evolution of the concept. We focus here on the major publications by Wenger et al. and other relevant articles published around the same period to explain the background of CoP.

### Lave and Wenger (1991)

In their earliest work, Lave and Wenger suggested that most of the learning for practitioners occurs in social relationships at the workplace rather than in a classroom setting, a concept known as 'situated learning'[16]. The central themes of this book are the interactions between novices and experts, and the process by which newcomers create a professional identity. To illustrate these themes, Lave and Wenger used the example of how midwives, meat cutters, and tailors learned their skills onsite in the environment where these skills were used. Much of the learning happened during informal gatherings where professionals interacted with each other and shared stories about their experience, and where novices consulted openly with experts. Through this process, gaps in the practice were identified and solutions were developed. The informal interactions eventually became the means for practitioners to improve practice and generate new ways to address recurrent problems[16].

The similarities between Lave and Wenger's viewpoint and the apprenticeship model of learning in the workplace are obvious. The challenges discussed in this publication are similar to those experienced by members in a learning community, including the tension and conflicts between novices and experts. In this book, CoP is loosely defined as people from the same discipline improving their skills by working alongside experts and being involved in increasingly complicated tasks. The journey from being a newcomer to becoming an expert is captured in the concept of 'legitimate peripheral learning,' in which newcomers are given opportunities to learn by engaging in simple tasks. Those who eventually master the skills become experts and subsequently assume the responsibility of mentoring other newcomers. In this context, CoPs can be viewed as a system for people to acquire and polish existing skills rather than to create new ways to complete a task[27].

A few issues were left unresolved in this work, however. Although the hierarchy of power between experts and novices is relatively clear, Lave and Wenger offered little insight into the potential for conflicts among experts or among novices[27]. Furthermore, although they stressed that CoPs cannot be purposefully formed by organizations, apprenticeship programs and clinical placements can be formally developed for mentoring new health professionals and trainees. It is unclear whether these programs still fit within the concept of CoP.

The view of 'learning on the job' is supported by Brown and Duguid's[28] 1991 publication, but in a slightly different way. They argued that all canonical (abstracted, orthodox, managerial) accounts of work were inflexible, impractical, and flawed, and that 'local understanding' of a problem was required to solve a problem and complete a task. As such, they used the CoP concept to describe how workers engage in informal groups both at work and off the job to share information and to develop new solutions for job-related problems. The latter deviated from Lave and Wenger's focus on existing skills, and moved on to the creation of new knowledge.

Brown and Duguid also focused on the close relationships among working, learning, and innovating for workers, and stressed the importance of the social environment in advancing practitioners' skills and knowledge in organizations. They encouraged interaction of workers across different communities within and outside of their own organisation, a concept known as 'community-of-communities' [28]. The underlying assumption of this work is that everyone involved is viewed as equal. However, in reality the dynamics among individuals are likely more complex, especially when one community has power over another (e.g., a manager community versus a technician community in the same organization), or when they are in direct competition. Furthermore, communities may have different goals, cultures, and politics, all of which may pose challenges for individuals who attempt to balance their participation across different communities[29]. Despite these issues, Brown and Duguid downplayed the potential conflicts, and their interpretation of the CoP concept might therefore have been overly optimistic.

### Wenger (1998)

Wenger used situated learning as his building block to expand the concept of the CoP in his 1998 book. He borrowed theoretical aspects from education, sociology, and social theory to refine the CoP concept, with a focus on socialization and learning, and the individual's identity development. His discussion was based on a case study of how medical claims processing clerks interact with each other and share information for doing routine office work. Instead of expanding the concept based on the novice-expert relationship, this book described CoP as an entity bounded by three interrelated dimensions: mutual engagement, joint enterprise, and a shared repertoire. 'Mutual engagement' represents the interaction between individuals that leads to the creation of shared meaning on issues or a problem. 'Joint enterprise' is the process in which people are engaged and working together toward a common goal. Finally, 'shared repertoire' refers to the common resources and jargons that members use to negotiate meaning and facilitate learning within the group. The three dimensions attempt to outline the process of individuals' interactions within CoP groups, but it is not clear what distinguishes them from other group structures. For example, members of a multidisciplinary care team work together to improve the health of their patients (i.e., joint enterprise), communicate with each other about patient care (i.e., mutual engagement), and develop ways and resources to adapt practice guidelines in their work (i.e., shared repertoires). In this case, it would not be unreasonable to argue that a multidisciplinary team that operates on these three axes is a CoPs[30]. However, it is less clear if the team is still a CoP if its internal communications are less than frequent, if team members rarely socialise with each other, and if half of the members do not use the available resources to improve practice.

Wenger's 1998 publication contains his first discussion of the importance of trajectories through different levels of participation within a group, and the tension of individuals belonging to multiple groups that are collaborating or competing, or have no relations with each other. In addition to the three dimensions, he also proposed 14 indicators for detecting the presence of a CoP, although most of them are rather abstract. These indicators are presented in Table 2 with our interpretation of the representative dimensions. Interestingly, most of these indicators focus on 'mutual engagement' and 'shared repertoire,' and only two (#2 and #7) appear to address the process of people working toward a common goal (i.e., joint enterprise). Attempts have been made to apply these indicators for the purpose of measurement, but because no validated measure has been used, the results are difficult to interpret[31].

**Table 2:** Wenger's indicators for the presence of community of practice and the proposed domains

**Wenger's indicators**	**CoP domains**
1. Sustained mutual relationships – harmonious or conflictual	Mutual engagement
2. Shared ways of engaging in doing things together	Mutual engagement Joint enterprise
3. The rapid flow of information and propagation of innovation	Mutual engagement
4. Absence of introductory preambles, as if conversations and interactions were merely the continuation of an ongoing process	Mutual engagement Shared repertoire
5. Very quick setup of a problem to be discussed	Mutual engagement Shared repertoire
6. Substantial overlap in participants' descriptions of who belongs	Mutual engagement
7. Knowing what others know, what they can do, and how they can contribute to an enterprise	Mutual engagement Joint enterprise Shared repertoire
8. Mutually defining identities	Mutual engagement
9. The ability to assess the appropriateness of actions and products	Shared repertoire
10. Specific tools, representations, and other artefacts	Shared repertoire
11. Local lore, shared stories, inside jokes, knowing laughter	Shared repertoire
12. Jargon and shortcuts to communication as well as the ease of producing new ones	Shared repertoire Mutual engagement
13. Certain styles recognized as displaying membership	Mutual engagement
14. A shared discourse reflecting a certain perspective on the world	Mutual engagement

* From: Wenger E. *Communities of Practice: Learning, Meaning, and Identity*. New York: Cambridge University Press; 1998, pg. 125.

The 1998 work also raised controversies about the use of the term 'community.' Contu and Willmott[32] pointed out that members of a CoP usually come together to address a problem or concern, but in reality not all communities are developed with a purpose. In this sense, the term 'community' could lead people to think that any group structure can be regarded as a CoP, which was not Wenger's intent. Overall, the depiction of the CoP in the 1998 publication is prone to a variety of interpretations and is challenging to apply.

In the late 1990s, reports about groups labelled as 'communities of practice' began to emerge in the literature. For example, Orr's ethnographic study, *Talking about Machine*, documented an example involving Xerox technicians who discovered specific trends of machine malfunctions through their frequent informal discussions and storytelling[33]. They eventually invented new ways to service the machines. Interestingly, instead of the term 'community of practice' Orr used 'occupational community,' which suggests a focus on the workers' ability to meet the company's goals (i.e., to service the machines) rather than the individuals' goals (e.g., professional growth and development)[27]. Other examples of CoPs include the community of automobile engineers at the Chrysler Corporation[34], the multidisciplinary community at the World Bank[5], and the multi-site online community at Caterpillar Inc[35]. CoPs are also widely used in the education[36,37] and information science[38,39] sectors. For example, Palincsar et al[36] described the process of developing an online CoP for science teachers in Michigan to share their knowledge of and experience in teaching kindergarten through Grade 5. A number of other online CoPs have also appeared in recent years [40-43], including the CP Square , which is a 'CoP of CoPs' hosted by Wenger and colleagues.

### Wenger, McDermott, and Snyder (2002)

In 2002 Wenger, McDermott, and Snyder authored *Cultivating Communities of Practice*[5]. In this book, the authors shifted their focus from individuals' learning and identity development on to providing a tool for organizations to manage 'knowledge workers.' In a marked departure from the previous publications, which suggested that CoP groups emerge spontaneously, this work suggested that organizations can engineer and cultivate CoPs to enhance their competitiveness[5,44]. Here CoP was vaguely defined as 'groups of people who share a concern, a set of problems, or a passion about a topic, and who deepen their knowledge and expertise in this area by interacting on an ongoing basis' (p. 4)[5]. This definition is even vaguer than the 14 indicators in Wenger's 1998 book, and although it does not limit CoP to groups within a company, the examples given are mainly from the business sector. Rather than centring on the performance of daily office work, this book portrayed CoP as the means to foster innovation and creative problem solving. Although the organization does not impose rules and regulations within a CoP, it can certainly influence the agenda and the composition of members.

To enable organizations to use CoP as a management tool, Wenger et al. revised the three characteristics of CoP and named them 'domain,' 'community,' and 'practice'[5,15,45]. The *domain *creates the common ground (i.e., the minimal competence that differentiates members from non-members) and outlines the boundaries that enable members to decide what is worth sharing and how to present their ideas. The *community *creates the social structure that facilitates learning through interactions and relationships with others. The *practice *is a set of shared repertoires of resources that include documents, ideas, experiences, information, and ways of addressing recurring problems. In essence, the practice is the specific knowledge the community shares, develops, and maintains. The authors claimed that CoPs can optimise the creation and dissemination of knowledge when the three elements work well together in a mature CoP; however, it was less clear on how to foster the three elements at the early stage.

Wenger et al. also introduced the roles of leaders/champions and facilitators[5]. Typically, the leader/champion is someone who is well respected within an organization, and often holds a leadership position. He/she is responsible for spreading the word about the group, recruiting members, and providing resources for group activities. The facilitator, on the other hand, is responsible for the group's day-to-day activities. This role is usually assumed by a senior manager who understands the overall mission of the organization, is resourceful, and is well connected with members and potential members of the CoP.

The involvement of a facilitator is perhaps one of the most frequently observed features in the subsequent studies of CoPs, some of which link the success or failure of the group to this role[7,13,35,46-53]. However, the actual responsibilities and the organizational support provided for this role vary across studies. For example, some facilitators play a distinct role from that of the leader and conduct their activities under the direction of the group and/or the leader[13,46,52], while other groups merge the role of the leader and facilitator[47,48]. The choice of management structure appears to depend on the size of the group and the availability of human resources. Which model best suits which type of organisation is unclear, but facilitator fatigue has been mentioned as something that can lead to the breakdown of CoP groups[47].

The 2002 book also attempted to compare the characteristics of CoP groups with other structures, although some components outlined by the authors are vague and contradictory. For example, they suggested that CoP groups are different from project teams because members of CoPs are self-selected and participation is voluntary. However, people from the same discipline or workplace automatically belong to the same CoP. Wenger et al. also said that CoPs are different from communities of interest, but others, like Fisher, argued that the latter can be a variation on a CoP since both can be identified by their *domain*, *community*, and *practice*[54]. The differences between the two types of communities are sufficiently vague for Fischer to claim that a CoP is a 'homogeneous community' consisting of members from a single discipline (e.g., physicians, researchers, or health care administrators), whereas a community of interest is a 'heterogeneous community' or 'community-of-communities' that mirrors a multidisciplinary team[54].

Other interpretations of CoP groups have emerged since the publication of this book. For example, Saint-Onge and Wallace described CoPs with three different components: 'people' (who is involved), 'practice' (what members do), and 'capabilities' (the ability to leverage competitive advantage in the business sector)[44]. Furthermore, they proposed three levels of CoPs based on the organizational structure and governance: 'informal groups' that aim to provide a forum for discussion among practitioners who are interested in a topic, 'supported groups' that are sponsored by the management and aim to build knowledge and skills for a given competency area, and 'structured groups' that are developed and managed by an organization and aim to advance the organization's business strategy[44]. The different interpretations of CoP make it challenging for people to apply this concept or to take full advantage of the benefits that CoP groups may offer. It is also difficult to objectively evaluate the effectiveness of these groups as there is no consensus on what is, or is not, a true CoP group.

## Conclusion

CoP is gaining popularity in health care, but the research in this area is relatively new and limited. Although the term began to surface in the literature in the mid-1990s, most primary studies were not published until 2000 or later. It should be noted that CoP was originally developed as a learning theory that promotes self-empowerment and professional development, but as the theory evolved, it became a management tool for improving an organization's competitiveness. The tension between satisfying individuals' needs for personal growth versus the organization's bottom line is perhaps the most contentious of the issues that make the CoP theory challenging to apply. Furthermore, as the definition broadens, it becomes more difficult to characterise what is and is not a CoP group. This potentially limits our ability to study CoPs as a strategy to improve clinical practice.

Because CoP is an evolving concept, it may be premature to set concrete boundaries to differentiate CoPs from other types of group structure. Nonetheless, the CoP concept can be used to provide some guidance for the development of groups, teams, and networks. Our analysis of the germinal literature highlighted several key characteristics of the CoP concepts, such as the support for formal and informal interaction between novices and experts, the emphasis on learning and sharing knowledge, and the investment to foster the sense of belonging among members. Hence, research in CoP may be more productive if we endeavor to develop and refine interventions that optimise these characteristics. Examples of promising interventions may include using a facilitator to promote network/group activities and enhance interaction among members[47], using information technology to facilitate communication of individuals in distributed networks/groups[52], or providing organizational infrastructures that promote the uptake of new knowledge in health care settings[55]. Furthermore, we believe that the functions of these network/groups may be optimized by improving the understanding of the process of negotiating boundaries of emerging CoPs, and the roles and responsibilities of CoP members.

## Competing interests

The authors declare that they have no competing interests.

## Authors' contributions

LCL, JMG, IDG developed the concept for the manuscript. LCL drafted the manuscript. All authors provided comments and approved the final version.
